# Presence and Onset of Chronic Kidney Disease as a Factor Involved in the Poor Prognosis of Patients with Essential Thrombocythemia

**DOI:** 10.1155/2024/9591497

**Published:** 2024-02-08

**Authors:** Yoshinori Hashimoto, Hiromi Omura, Takayuki Tanaka

**Affiliations:** Department of Hematology, Tottori Prefectural Central Hospital, Tottori, Japan

## Abstract

Chronic kidney disease (CKD) is an important risk factor for cardiovascular disease, thrombosis, and all-cause death. However, few studies have examined the association between CKD and the prognosis of patients with essential thrombocythemia (ET). We collected ET patients who met the WHO classification 2017 and performed a retrospective clinical study to clarify the association between the presence and onset of CKD and prognosis. Of 73 patients who met the diagnostic criteria, 21 (28.8%) had CKD at the time of ET diagnosis. The age of patients with CKD was significantly higher, and a high proportion of these patients had the *JAK2*V617F gene mutation. The presence of CKD was a risk factor for the prognosis (hazard ratio (HR): 3.750, 95% confidence interval (CI): 1.196–11.760, *P*=0.023), and the survival curve was significantly poorer. Furthermore, we analyzed patients without CKD at the time of ET diagnosis using the onset of CKD as a time-dependent variable and identified the onset of CKD as a risk factor for the prognosis (HR: 9.155, 95% CI: 1.542–54.370, *P*=0.005). In patients with renal hypofunction at the time of ET diagnosis or those with a reduction in the kidney function during follow-up, strict renal function monitoring at regular intervals is necessary.

## 1. Introduction

Essential thrombocythemia (ET) is a subtype of Philadelphia chromosome-negative myeloproliferative neoplasms (MPNs). It is characterized by mature megakaryocytes in the bone marrow and peripheral blood thrombocytosis [1]. The prognosis of patients with ET is poor in comparison with the general population [2]. Currently, in clinical practice, as a risk classification for prognosis, the International Prognostic Score for Essential Thrombocythemia (IPSET) model involving 3 factors: an age of ≥60 years, leukocytosis, and a history of thrombosis is routinely used, and its usefulness has been reported [3]. After the *JAK2*V617F gene mutation was found in 2005 [4, 5], there was no large-scale cohort study involving ET patients in Japan. However, a retrospective study (JSH-MPN-18 study) was conducted under the leadership of the Japanese Society of Hematology, and the validity of the IPSET model was also confirmed in Japanese patients [6]. In addition, recently, a risk classification for prognosis in consideration with nondriver gene mutations was developed [7], and prognostic factors have been energetically identified and analyzed.

Chronic kidney disease (CKD) is closely associated with cardiovascular risk factors, such as hypertension, diabetes mellitus, and dyslipidemia, being an important risk factor for cardiovascular disease, thrombosis, and all-cause death in the general population [8–10]. Previous studies found the association between cardiovascular risk factors and thrombosis in patients with MPNs [11, 12], but few studies have examined the association between CKD and the onset of thrombosis or prognosis [13, 14]. Furthermore, a study investigated serial changes in the estimated glomerular filtration rate (eGFR) during the observation period in patients with ET [15–17]. However, no study has examined the prognosis of patients who developed CKD during follow-up.

As the number of patients with CKD has been increasing, it is important to evaluate the influence of the presence of CKD, ET itself, or ET treatment-related progression of renal dysfunction on the prognosis of patients for establishing future ET treatment approaches. The purpose of this study was to clarify whether CKD at the time of ET diagnosis is a prognostic factor, whether the onset of CKD during the course is a prognostic factor in ET patients without CKD at the time of diagnosis, and risk factors for the onset of CKD.

## 2. Materials and Methods

### 2.1. Patients and Methods

We examined ET patients who consulted our hospital between April 2008 and December 2022 and were definitively diagnosed through reevaluation based on the diagnostic criteria described in the WHO classification 2017 [18]. The patients included those in whom polycythemia vera (PV) or primary myelofibrosis (PMF) (especially PMF, prefibrotic/early stage) was suspected from the peripheral blood data, but a diagnosis of ET was histologically made. For analysis, the following clinical parameters and events were adopted: the date of diagnosis, age, sex, white blood cell count, percent neutrophils, red blood cell count (RBC), hemoglobin (Hb), hematocrit, platelet count, C-reactive protein (CRP), lactate dehydrogenase (LD), blood urea nitrogen, creatinine (Cr), eGFR, uric acid (UA), presence or absence of driver gene mutations, presence or absence of chromosomal abnormalities, history of thrombosis, history of hemorrhagic events, presence or absence of cardiovascular risk factors (hypertension, diabetes mellitus, dyslipidemia, and smoking), presence or absence of heart failure, presence or absence of splenomegaly, presence or absence of treatment/its details, changes in eGFR after ET diagnosis, presence or absence of thrombosis/hemorrhagic events/disease transformations/secondary cancers after ET diagnosis, and outcome (all-cause death and its cause). This study was performed according to the principles of the Declaration of Helsinki and was approved by the Ethics Committee of Tottori Prefectural Central Hospital (Approval No. 2023-22).

### 2.2. Definitions

For the risk classification of prognosis, the IPSET model was used [3]. Thromboses were defined as cerebral infarction, transient ischemic attacks, myocardial infarction, angina pectoris, peripheral artery occlusion, pulmonary embolism, deep venous thrombosis, and other life-threatening types of thrombosis. Hemorrhagic events were defined as cerebral hemorrhage, gastrointestinal hemorrhage, and other life-threatening hemorrhagic events. Patients who met the diagnostic criteria for post-ET myelofibrosis or acute myeloid leukemia according to the international consensus classification of myeloid neoplasms and acute leukemias [19] were regarded as showing disease transformation. Secondary cancers were defined as new malignancies that developed during follow-up regardless of the presence or absence of drug usage.

As an index of the kidney function, eGFR was used. For the calculation of eGFR, the kidney function of Japanese patients may be overestimated using the Modification of Diet in Renal Disease (MDRD) formula [20], which is internationally used; therefore, we used the Japanese Society of Nephrology (JSN) eGFR in this study: JSN eGFR (mL/min/1.73 m^2^) = 194 × (serum Cr value) − 1.094 × (Age)^−0.287^ (if female × 0.739) [21]. Concerning the definition of CKD [22], the number of patients in whom urinalysis had been performed at the time of ET diagnosis and during follow-up was limited, and CKD was defined as a case in which an eGFR of <60 mL/min/1.73 m^2^ had persisted for >3 months (eGFR had been measured twice or more during a >3-month period). Regarding the onset of CKD during the observation period, a point when eGFR had met the criterion for >3 months was regarded as the date of diagnosis.

Overall survival (OS) was calculated as the term from the day of diagnosis to the end of observation, and discontinuation of the patient visit and death were considered censored events.

### 2.3. Statistical Analysis

We described the background and events divided into two groups: those with and without CKD at the time of ET diagnosis. Nominal variables were analyzed using Fisher's exact test or the chi-square test and continuous variables were analyzed using the Mann–Whitney *U* test. Survival curves were plotted using the Kaplan–Meier method. For comparison, the log-rank test was used. To identify risk factors for death, thrombosis, hemorrhagic events, disease transformation, and onset of CKD, uni-/multivariate analyses by Cox proportional hazard regression were performed. Variables that were significant in univariate analysis were selected as variables in multivariate analysis. Concerning patients who newly developed CKD after ET diagnosis, the onset of CKD was used as a time-dependent variable. As described above, uni-/multivariate analyses of factors that significantly influence OS were performed using Cox proportional hazard regression. Clinically important variables were selected based on previous studies [3, 6, 11] and included for analysis in this study without using a stepwise selection. For statistical analysis of valid variables, two-sided tests were performed, and a *P* value of 0.05 was regarded as significant. We used EZR version 1.55 (Jichi Medical University, Saitama Medical Center, Japan) statistical software [23]. EZR is a graphical user interface for R (The R Foundation for Statistical Computing, Vienna, Austria).

## 3. Results

### 3.1. Patient Characteristics

Of 73 patients who met the diagnostic criteria, 21 (28.8%) had CKD at the time of ET diagnosis (Table 1). We compared the patient background between patients with and without CKD at the time of ET diagnosis. In the former, the age, RBC, LD, and UA levels were higher. In addition, the proportion of patients with the *JAK2*V617F gene mutation was higher. Furthermore, the proportion of patients with cardiovascular risk factors or comorbidities, such as heart failure, was higher. The median observation period was 4.8 years, and the 5-year survival rate was 84.0% (95% confidence interval [CI] 71.0–91.5%). Concerning events after ET diagnosis, thrombosis occurred in 10 patients (13.7%), hemorrhagic events in 9 (12.3%), disease transformations in 6 (8.2%), and secondary cancers in 9 (12.3%). Thirteen patients (17.8%) died (Supplemental Table 1). In the patients with CKD, the mortality was higher, and the primary causes of death included infectious diseases and heart failure.

### 3.2. Presence of CKD as a Poor Prognostic Factor in ET Patients

**Figure 1:**
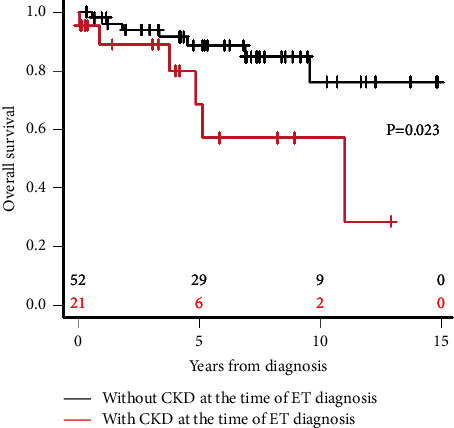
Comparison of survival curves between the two groups with (red) and without CKD (black) at the time of ET diagnosis. The *P* value was generated by the log-rank test.

To investigate whether the presence of CKD at the time of diagnosis is a risk factor for the prognosis of patients with ET, we performed uni-/multivariate analyses of OS. As shown in Table 2, on univariate analysis, a CRP level of ≥0.30 mg/dL (hazard ratio (HR) 3.620, 95% CI 1.098–11.980, *P*=0.035), CKD presence (HR 3.332, 95% CI 1.113–9.974, *P*=0.031), and splenomegaly (HR 3.680, 95% CI 1.234–10.970, *P*=0.019) were extracted as risk factors. Multivariate analysis showed that CKD presence (HR 3.750, 95% CI 1.196–11.760, *P*=0.023) and splenomegaly (HR 4.304, 95% CI 1.371–13.520, *P*=0.012) were both independent risk factors for the prognosis. Furthermore, the OS in the patients with CKD at the time of ET diagnosis was significantly poorer than that in those without CKD (Figure 1).

### 3.3. Onset of CKD during the Observation Period as a Poor Prognostic Factor in ET Patients

We divided 52 patients without CKD at the time of ET diagnosis into two groups: those with and without the onset of CKD during the observation period, and compared their characteristics (Supplemental Table 2). Eight patients (15.4%) developed CKD during the observation period. There were no significant differences in the median age or presence of cardiovascular risk factors between the two groups. However, in the CKD onset group, the median Hb level was significantly lower, and the median platelet count was higher. Uni-/multivariate analyses of OS were performed using the onset of CKD as a time-dependent variable in addition to conventional variables (Table 3). Univariate analysis revealed a platelet count of ≥1000 × 10^9^/L, splenomegaly, and CKD onset were extracted as risk factors for OS. Multivariate analysis showed that only the onset of CKD (HR 9.155, 95% CI 1.542–54.370, *P*=0.005) was a risk factor. Risk factors for the onset of CKD were the presence of chromosomal abnormalities and use of anagrelide (Supplemental Table 3). Of the patients who developed CKD, thrombosis was noted in 1 after diagnosis, hemorrhage in 1, and disease transformation in 3 (no event: 3 patients). However, there was no direct association with renal hypofunction.

## 4. Discussion

**Figure 2:**
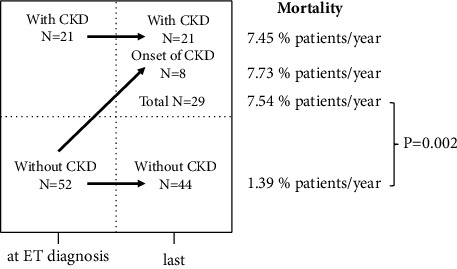
A schematic representation of the development status of CKD and mortality from ET diagnosis to last follow-up. The *P* value was generated by the log-rank test.

This is the first report to demonstrate that the prognosis of patients with CKD at the time of ET diagnosis is poor among Japanese patients with ET and that the new onset of CKD during the observation period leads to a poor prognosis by focusing on time-dependent variables. In brief, patients who ultimately suffered from CKD had a significantly poorer prognosis than those without groups (Figure 2) (*P*=0.002).

Since the entity “MPN-related glomerulopathy” was reported in 2011 [24], the association between MPNs and renal hypofunction has attracted attention. The incidence of CKD at the time of ET diagnosis is reportedly higher than in the general population [25, 26], being 12.9 to 28.9% [13, 15–17, 27, 28]. The incidence of CKD in this cohort (28.8%) was similar to that in Europe, although it is slightly lower at approximately 15% in Asians. As the reason for this, our cohort reflected the influence of a high-median-age population based on real-world data from actual clinical practice. Characteristics of ET patients with CKD included not only older age but also a significantly higher proportion of the patients with the *JAK2*V617F gene mutation. This has not been noted in a combined analysis of ET and PV patients or in a cohort consisting of PV or PMF patients alone [13, 27]. This finding may be characteristic of ET patients.

Previous studies on MPNs demonstrated that the presence of CKD at the time of diagnosis in ET and PV patients was a risk factor for thrombosis [13]. Furthermore, it has been shown that the presence of CKD at the time of diagnosis in PMF patients is a risk factor for thrombosis and prognosis [14]. On the other hand, few studies have examined the relationship between CKD and prognosis in patients with ET. A recent study found that a Cr level of >0.9 mg/dL was a poor prognostic factor in ET patients [27]. In the above study, the serum Cr level was measured once several years after MPN diagnosis. This parameter is influenced by the muscle volume; therefore, there may be a bias. This study comparing survival curves between the two groups with respect to the presence or absence of CKD at the time of ET diagnosis and multivariate analysis of OS suggested that the presence of CKD is a risk factor for the prognosis of patients with ET.

Some studies have often focused on the linear regression coefficient of eGFR as an observation item during the course [15–17]. A study reported that there was a rapid reduction in the kidney function in 20% of patients with MPNs, whereas an improvement in the kidney function was achieved in approximately 50% [15]. Furthermore, it was suggested that the eGFR slope is poor in MF patients or under the use of anagrelide [15, 17, 28], whereas it is good in PV patients or under the use of hydroxyurea [16, 29]. However, no study has shown a direct association between the new onset of CKD and prognosis. We reported that the new onset of CKD was a risk factor for the prognosis using the onset of CKD as a time-dependent variable. As a factor for renal hypofunction, the use of anagrelide was suggested, as previously reported [28]. This may be because dilation of the afferent glomerular arteriole related to inhibitory actions on PDE3 results in an increase in the intraglomerular pressure. However, concerning some drugs, there was a reduction in eGFR in the initial phase of administration, whereas the eGFR slope became gentler thereafter, as shown for losartan [30] and sodium glucose cotransporter 2 inhibitors [31]. Long-term follow-up data are necessary.

As the limitations of this study, this was a single-center retrospective study involving a small number of patients, and the JSN eGFR was used for renal function assessment; our data cannot be simply compared with data from Europe or the United States. In addition, we cannot rule out the possibility that extra renal factors may have influenced the calculation of eGFR. For CKD diagnosis, urinalysis was not taken into consideration. However, the accuracy and quality of this study are secured.

In conclusion, the results suggested that the presence of CKD at the time of ET diagnosis and onset of CKD during follow-up are prognostic factors. In patients with renal hypofunction at the time of ET diagnosis or those with a reduction in the kidney function during follow-up, close kidney function monitoring at regular intervals is necessary. Furthermore, a prospective observational study should be performed to investigate whether treatment approaches, such as cytoreductive therapy, contribute to an improvement in the prognosis, regarding renal hypofunction as an MPN-related symptom.

## Figures and Tables

**Table 1:** Patient characteristics.

Characteristics at ET diagnosis	All patients *n* = 73	With CKD at ET diagnosis *n* = 21 (28.8%)	Without CKD at the time of ET diagnosis *n* = 52 (71.2%)	*P* value
Age, median; years (range)	69 (29–89)	77 (45–89)	68 (29–89)	**0.001**
Female, *n* (%)	38 (52)	9 (43)	29 (56)	0.438
Male, *n* (%)	35 (48)	12 (57)	23 (44)	
WBC, median; ×10^9^/L (range)	9.6 (4.0–48.6)	10.3 (7.3–35.8)	9.1 (4.0–48.6)	0.070
Neu, median; % (range)	70.8 (50.1–90.3)	71.7 (53.0–90.3)	70.8 (50.1–88.9)	0.446
RBC, median; ×10^12^/L (range)	4.82 (2.54–6.72)	5.29 (2.54–6.72)	4.71 (3.05–6.72)	**0.027**
Hb, median; g/dL (range)	14.0 (7.5–16.4)	14.1 (7.5–16.4)	13.9 (7.8–16.1)	0.869
Hct, median; % (range)	43.0 (23.6–54.6)	43.9 (23.6–54.6)	42.6 (25.6–49.2)	0.249
Plt, median; ×10^9^/L (range)	879 (450–2648)	889 (452–2648)	868 (450–1833)	0.942
CRP median; mg/dL (range)	0.07 (0.01–4.52)	0.13 (0.02–3.63)	0.07 (0.01–4.52)	0.093
LD, median; U/L (range)	239 (123–609)	273 (179–561)	214 (123–609)	**0.002**
BUN, median; mg/dL (range)	14.4 (6.9–37.6)	19.0 (9.5–37.6)	13.7 (6.9–26.2)	**0.002**
Cr, median; mg/dL (range)	0.7 (0.4–2.1)	1.0 (0.7–2.1)	0.6 (0.4–0.9)	**<0.001**
eGFR, median; ml/min/1.73 m^2^ (range)	80.2 (25.2–133.9)	52.3 (25.2–59.5)	87.1 (62.6–133.9)	**<0.001**
Uric acid, median; mg/dL (range)	5.1 (2.3–8.7)	6.6 (3.7–8.5)	4.8 (2.3–8.7)	**<0.001**
*JAK2*V617F mutation, *n* (%)	49^a^ (67.1)	19 (90.5)	30^a^ (57.7)	**0.007**
*CALR* mutation, *n* (%)	12 (16.4)	2 (9.5)	10 (19.2)	0.489
*MPL* mutation, *n* (%)	4^a^ (5.5)	0 (0)	4^a^ (7.7)	0.318
Chromosome abnormality, *n* (%)	4 (5.5)	2 (9.5)	2 (3.8)	0.403
History of thrombosis, *n* (%)	22 (30.1)	10 (47.6)	12 (23.1)	0.051
History of hemorrhagic events, *n* (%)	4 (5.5)	1 (4.8)	3 (5.8)	1.000
Cardiovascular risk factors, *n* (%)	48 (65.8)	18 (85.7)	30 (57.7)	**0.029**
Heart failure, *n* (%)	13 (17.8)	8 (61.9)	5 (9.6)	**0.007**
Splenomegaly, *n* (%)	13 (17.8)	4 (19.0)	9 (17.3)	1.000

CKD, chronic kidney disease; eGFR, estimated glomerular filtration rate; ET, essential thrombocythemia. *P* values of <0.05 are highlighted in bold. Percentages in parentheses refer to percentages in each group. ^a^One patient harbored *JAK2*V617F and *MPL*W515L mutations.

**Table 2:** Univariable and multivariable analyses of predictors of overall survival.

Variables	Univariable			Multivariable		
	HR	95% CI	*P* value	HR	95% CI	*P* value
Age ≥60 years	2.203	0.488–9.956	0.305			
Gender (male)	1.780	0.589–5.381	0.307			
WBC ≥11 × 10^9^/L	1.305	0.437–3.899	0.634			
Plt ≥1000 × 10^9^/L	2.157	0.722–6.443	0.169			
CRP ≥0.30 mg/dL	3.620	1.098–11.980	**0.035**	2.830	0.837–9.566	0.094
Uric acid ≥7.0 mg/dL	1.595	0.342–7.444	0.552			
CKD	3.332	1.113–9.974	**0.031**	3.750	1.196-11.760	**0.023**
*JAK2*V617F mutation	2.546	0.560–11.570	0.226			

*Chromosome abnormality*	*All patients with chromosome abnormalities survived*					
History of thrombosis	2.636	0.873–7.962	0.086			
History of hemorrhagic events	1.470	0.188–11.490	0.714			
Cardiovascular risk factors	2.351	0.638–8.672	0.199			
Splenomegaly	3.680	1.234–10.970	**0.019**	4.304	1.371-13.520	**0.012**
Antiplatelet therapy	1.214	0.153–9.639	0.854			
Cytoreductive therapy with HU	0.700	0.212–2.311	0.558			
Cytoreductive therapy with ANA	0.773	0.167–3.577	0.742			

ANA, anagrelide; CI, confidence interval; CKD, chronic kidney disease; HU, hydroxyurea; HR, hazard ratio. *P* values of <0.05 are highlighted in bold.

**Table 3:** Univariable and multivariable analyses of predictors of overall survival in patients without chronic kidney disease at the time of ET diagnosis.

Variables	Univariable			Multivariable		
	HR	95% CI	*P* value	HR	95% CI	*P* value
Age ≥60 years	3.402	0.409–28.290	0.257			
Gender (male)	4.027	0.776–20.890	0.097			
WBC ≥11 × 10^9^/L	1.370	0.304–6.181	0.682			
Plt ≥1000 × 10^9^/L	6.015	1.147–31.55	**0.034**	2.735	0.322–23.210	0.357
CRP ≥0.30 mg/dL	4.786	0.961–23.830	0.056			
Uric acid ≥7.0 mg/dL	2.570	0.298–22.180	0.391			
*JAK2*V617F mutation	1.644	0.317–8.519	0.554			

*Chromosome abnormality*	*All patients with chromosome abnormalities survived*					
History of thrombosis	2.750	0.613–12.340	0.187			
History of hemorrhagic events	2.493	0.290–21.440	0.406			
Cardiovascular risk factors	2.262	0.431–11.870	0.335			
Splenomegaly	5.452	1.219–24.390	**0.026**	2.809	0.420–18.780	0.287
Antiplatelet therapy	0.586	0.068–5.071	0.627			
Cytoreductive therapy with HU	0.337	0.061–1.843	0.209			
Cytoreductive therapy with ANA	1.459	0.267–7.974	0.663			
Onset of CKD	11.550	2.074–64.280	**0.005**	9.155	1.542–54.370	**0.015**

ANA, anagrelide; CI, confidence interval; CKD, chronic kidney disease; ET, essential thrombocythemia; HU, hydroxyurea; HR, hazard ratio. *P* values of <0.05 are highlighted in bold.

## Data Availability

The data used to support the findings of the study are available from the corresponding author upon request.

## Supplementary Materials

Supplementary Materials The supplementary content includes details of events after ET diagnosis (Supplemental Table 1), characteristics between the two groups with and without the onset of chronic kidney disease during the observation period (Supplemental Table 2), and analysis of predictors for the onset of chronic kidney disease during the observation period (Supplemental Table 3).
