# Supplementary material for: Presence and Onset of Chronic Kidney Disease as a Factor Involved in the Poor Prognosis of Patients with Essential Thrombocythemia
Source: Adv Hematol. 2024 Feb 8;2024:9591497. doi: 10.1155/2024/9591497 (PMC10869185; doi:10.1155/2024/9591497)
Supplement: Supplementary Materials — The supplementary content includes details of events after ET diagnosis (Supplemental Table 1), characteristics between the two groups with and without the onset of chronic kidney disease during the observation period (Supplemental Table 2), and analysis of predictors for the onset of chronic kidney disease during the observation period (Supplemental Table 3). [file 9591497.f1.docx]

**Supplemental Table 1** Post-diagnosis events

| Post-diagnosis Events | All patients  n=73 | With CKD  at ET diagnosis  n=21 (28.8%) | Without CKD  at ET diagnosis  n=52 (71.2%) |
| --- | --- | --- | --- |
| **Thrombosis, n (%), [% patients/year]** | **10 (13.7), [2.65]** | **2 (9.5), [2.62]** | **8 (15.4), [2.65]** |
| Arterial, n | 8 | 2 | 6 |
| Venous, n | 2 | 0 | 2 |
| **Hemorrhagic events, n (%), [% patients/year]** | **9 (12.3), [2.39]** | **4 (19.0), [5.03]** | **5 (9.6), [1.68]** |
| Cerebral hemorrhage, n | 3 | 1 | 2 |
| Gastrointestinal hemorrhage, n | 3 | 1 | 2 |
| Others, n | 3 | 2 | 1 |
| **Disease transformations, n (%), [% patients/year]** | **6 (8.2), [1.52]** | **0 (0), [0]** | **6 (11.5), [1.90]** |
| Secondary myelofibrosis, n | 4 | 0 (0) | 4 |
| Acute myeloid leukemia, n | 2 | 0 (0) | 2 |
| **Secondary cancers, n (%), [% patients/year]** | **9 (12.3), [2.35]** | **2 (9.5), [2.83]** | **7 (13.5), [2.24]** |
| **Death, n (%), [% patients/year]** | **13 (17.8), [3.19]** | **6 (28.6), [7.45]** | **7 (13.5), [1.93]** |
| Infectious diseases, n | 3 | 3 | 0 |
| Heart failure, n | 3 | 2 | 1 |
| Secondary cancers, n | 2 | 0 | 2 |
| Hemorrhagic events, n | 2 | 1 | 1 |
| Disease transformations, n | 1 | 0 | 1 |
| Others, n | 2 | 0 | 2 |

*CKD*, chronic kidney disease; *ET*, essential thrombocythemia

Percentages in parentheses refer to percentages in each group.

**Supplemental Table 2** Comparison of characteristics between the two groups with and without the onset of chronic kidney disease during the observation period

| Characteristics at ET diagnosis | Patients without CKD at ET diagnosis  n=52 | Developed CKD during follow-up  n=8 (15.4%) | Without CKD during follow-up  n=44 (84.6%) | P value |
| --- | --- | --- | --- | --- |
| Age, median; years (range) | 68 (29-89) | 71 (55-89) | 67 (29-86) | 0.191 |
| Female, n (%) | 29 (56) | 6 (75) | 23 (52.3) | 0.278 |
| Male, n (%) | 23 (44) | 2 (25) | 21 (47.7) |  |
| WBC, median; ×10^9^/L (range) | 9.1 (4.0-48.6) | 10.0 (6.7-48.6) | 8.7 (4.0-17.7) | 0.214 |
| Neu, median; % (range) | 70.8 (50.1-88.9) | 75.3 (54.7-88.9) | 69.6 (50.1-87.0) | 0.163 |
| RBC, median; ×10^12^/L (range) | 4.71 (3.05-6.72) | 4.41 (3.05-6.72) | 4.71 (3.90-5.79) | 0.145 |
| Hb, median; g/dL (range) | 13.9 (7.8-16.1) | 12.2 (7.8-14.3) | 14.0 (10.6-16.1) | **0.026** |
| Hct, median; % (range) | 42.6 (25.6-49.2) | 41.3 (25.6-44.5) | 42.6 (34.7-9.2) | 0.171 |
| Plt, median; ×10^9^/L (range) | 868 (450-1833) | 1112 (792-1636) | 831 (450-1833) | **0.030** |
| CRP median; mg/dL (range) | 0.07 (0.01-4.52) | 0.14 (0.02-3.41) | 0.07 (0.01-4.52) | 0.182 |
| LD, median; U/L (range) | 214 (123-609) | 239 (181-439) | 210 (123-609) | 0.264 |
| BUN, median; mg/dL (range) | 13.7 (6.9-26.2) | 14.2 (7.8-25.0) | 13.5 (6.9-26.2) | 0.478 |
| Cr, median; mg/dL (range) | 0.6 (0.4-0.9) | 0.5 (0.5-0.9) | 0.6 (0.4-0.9) | 0.183 |
| eGFR, median; ml/min/1.73m^2^ (range) | 87.1 (62.6-133.9) | 83.2 (62.6-118.2) | 87.3 (64.6-133.9) | 0.551 |
| Uric acid, median; mg/dL (range) | 4.8 (2.3-8.7) | 4.4 (3.6-8.7) | 4.8 (2.3-8.0) | 0.859 |
| *JAK2*V617F mutation, n (%) | 30^a^ (57.7) | 5 (62.5) | 25 (56.8) | 1.000 |
| *CALR* mutation, n (%) | 10 (19.2) | 2 (25.0) | 8 (18.2) | 0.642 |
| *MPL* mutation, n (%) | 4^a^ (7.7) | 1 (12.5) | 3 (6.8) | 0.499 |
| Chromosome abnormality, n (%) | 2 (3.8) | 1 (12.5) | 1 (2.3) | 0.287 |
| History of thrombosis, n (%) | 12 (23.1) | 2 (25.0) | 10 (22.7) | 1.000 |
| History of hemorrhagic events, n (%) | 3 (5.8) | 1 (12.5) | 2 (4.5) | 0.401 |
| Cardiovascular risk factors, n (%) | 30 (57.7) | 7 (87.5) | 23 (52.3) | 0.118 |
| Heart failure, n (%) | 5 (9.6) | 2 (25.0) | 3 (6.8) | 0.164 |
| Splenomegaly, n (%) | 9 (17.3) | 3 (37.5) | 6 (13.6) | 0.130 |

*CKD*, chronic kidney disease; *eGFR*, estimated glomerular filtration rate; *ET*, essential thrombocythemia

P value of <0.05 are highlighted in bold.

Percentages in parentheses refer to percentages in each group.

^a^One patient harbored *JAK2*V617F and *MPL*W515L mutations.

**Supplemental Table 3** Univariable and multivariable analyses of predictors for the onset of chronic kidney disease during the observation period

|  | Univariable | | |  | Multivariable | | |
| --- | --- | --- | --- | --- | --- | --- | --- |
| Variables | HR | 95% CI | P value |  | HR | 95% CI | P value |
| Age ≥ 60 years | 1.721 | 0.347-8.533 | 0.506 |  |  |  |  |
| Gender (male) | 0.439 | 0.088-2.174 | 0.313 |  |  |  |  |
| WBC ≥ 11×10^9^/L | 0.953 | 0.228-3.996 | 0.948 |  |  |  |  |
| Plt ≥ 1000×10^9^/L | 3.423 | 0.818-14.330 | 0.092 |  |  |  |  |
| CRP ≥ 0.30mg/dL | 2.842 | 0.678-11.910 | 0.153 |  |  |  |  |
| Uric acid ≥ 7.0mg/dL | 4.395 | 0.885-21.820 | 0.070 |  |  |  |  |
| *JAK2*V617F mutation | 1.307 | 0.312-5.472 | 0.714 |  |  |  |  |
| Chromosome abnormality | 14.660 | 1.299-165.400 | **0.030** |  | 44.960 | 2.958-683.300 | **0.006** |
| History of thrombosis | 1.172 | 0.236-5.813 | 0.846 |  |  |  |  |
| History of hemorrhagic events | 1.803 | 0.221-14.730 | 0.582 |  |  |  |  |
| Cardiovascular risk factors | 5.678 | 0.695-46.530 | 0.105 |  |  |  |  |
| Splenomegaly | 2.696 | 0.643-11.300 | 0.175 |  |  |  |  |
| Antiplatelet therapy | 1.003 | 0.123-8.174 | 0.998 |  |  |  |  |
| Cytoreductive therapy with HU | 0.212 | 0.043-1.055 | 0.058 |  |  |  |  |
| Cytoreductive therapy with ANA | 6.013 | 1.431-25.260 | **0.014** |  | 9.136 | 1.765-47.300 | **0.008** |

*ANA*, anagrelide; *CI*, confidence interval; *HU*, hydroxurea; *HR*, hazard ratio

P value of <0.05 are highlighted in bold.
